# *Phlebovirus* seroprevalence in Austrian Army personnel returning from missions abroad

**DOI:** 10.1186/s13071-019-3674-6

**Published:** 2019-08-24

**Authors:** Edwin Kniha, Adelheid G. Obwaller, Gerhard Dobler, Wolfgang Poeppl, Gerhard Mooseder, Julia Walochnik

**Affiliations:** 1 Institute of Specific Prophylaxis and Tropical Medicine, Center for Pathophysiology, Infectiology and Immunology, Medical University of Vienna, Vienna, Austria; 2 Federal Ministry of Defence, Division of Science, Research and Development, Vienna, Austria; 3 Bundeswehr Institute of Microbiology (BwIM), Munich, Germany; 4 Department of Infectious Diseases and Tropical Medicine, Medical University of Vienna, Vienna, Austria; 5 Department of Dermatology and Tropical Medicine, Military Medical Cluster East, Austrian Armed Forces, Vienna, Austria

**Keywords:** Sand fly fever, Military, Sand fly, Risk factors, Balkans, Middle East

## Abstract

**Background:**

Phleboviruses are mainly transmitted by sand flies and infections can result in various symptoms, including meningitis and meningoencephalitis. In endemic regions, seroprevalences in humans and animals are high. Military personnel on missions in endemic areas are at increased risk of infection, however, for soldiers from central European countries, data are scarce. The aims of this study were to determine the exposure to phleboviruses of Austrian soldiers returning from missions abroad and to assess potential risk factors. A retrospective serological study was performed with sera of 753 healthy Austrian soldiers returning from missions in Bosnia and Herzegovina (BIH, *n *= 61), Kosovo (*n *= 261), Syria (*n *= 101) and Lebanon (*n *= 63) and of soldiers prior to their missions (*n *= 267).

**Results:**

Altogether, 119 sera (15.8%, 119/753) were positive for anti-*Phlebovirus* IgG antibodies, with highest seroprevalences found in soldiers returning from Kosovo (20.69%, 54/261), followed by Syria (17.82%, 18/101), Lebanon (14.29%, 9/63) and BIH (11.48%, 7/61). Of the soldiers tested prior to their missions 11.61% (31/267) were positive. Of the 119 seropositive individuals, 30 (25.2%, 30/119) also had anti-*Phlebovirus* IgM antibodies. *Phlebovirus* seropositivity significantly correlated with symptoms of febrile illness during the respective mission (OR: 1.9, 95% CI: 1.1–3.4, *P *= 0.03) and with *Leishmania* seropositivity (OR: 2.7, 95% CI: 1.2–5.8, *P *= 0.009). Also, the outdoor activity “running” during the mission showed a strong trend towards an association with *Phlebovirus* seropositivity (OR: 1.9, 95% CI: 0.9–4.4, *P *= 0.08), and seropositivity generally increased with the duration of a mission (OR: 2.5, 95% CI: 0.9–7.5, *P *= 0.07).

**Conclusions:**

This study indicates that soldiers are exposed to sand flies and at significant risk for *Phlebovirus* infection during missions in the Mediterranean area and the Middle East. Adequate prevention measures should be applied particularly during vespertine outdoor activities.

## Background

Phleboviruses are transmitted by either sand flies, mosquitoes or ticks, with sand flies being the main vectors [1]. Among various described species, Toscana virus (TOSV), Sand fly fever Naples phlebovirus (SFNV), Sand fly fever Sicilian virus (SFSV) and Cyprus virus (CYPV), all occurring in the Mediterranean regions of Europe, Africa and Asia, are of highest medical relevance. *Phlebovirus* infections often remain asymptomatic, but can result in febrile illness with sudden high fever, headache, photophobia, malaise and retro-orbital pain, symptoms usually declining after a few days [2]. However, Toscana virus shows a strong neurotropism and is assumed to be majorly responsible for meningitis and meningoencephalitis during the summer months in Italy [2]. Further reports from endemic countries such as Portugal [3], France [4] and Spain [5] and an imported case in Switzerland [6] corroborate Toscana virus being an important cause of meningitis in Mediterranean countries. To date, no vaccine or specific drugs are available, but assumedly lifelong immunity to the respective *Phlebovirus* serotype is established after infection [7].

Sand fly fever has been known to be of considerable medical importance in military personnel for decades [8], particularly when large numbers of immunologically naïve soldiers are introduced to *Phlebovirus-*endemic regions [9]. Symptomatic as well as asymptomatic infections in military troops have been reported from various regions [10]. Outbreaks of sand fly fever have occurred in military personnel operating in Cyprus and Iraq [11, 12]. UK military personnel was shown to be exposed to sand flies and phleboviruses in Afghanistan and high numbers of sand fly fever virus infections were reported from military personnel deployed in Iran [13, 14].

The Austrian Armed Forces operate in several sand fly-endemic areas. Here, a retrospective serological study was performed in order to evaluate *Phlebovirus* seroprevalences among healthy Austrian soldiers returning from sand fly-endemic operational areas.

## Methods

### Serum samples

Altogether, 753 sera, all taken during routine medical examinations, were included in this study. Study participants showed a minimum age of 18 and a maximum age of 63 years (mean ± SD, 29.6 ± 10.04). Of the 753 participants, 747 were male and 6 were female; 486 sera derived from healthy soldiers after their return from missions abroad, i.e. 61 samples from soldiers returning from BIH, 101 from Syria and 63 from Lebanon, all collected in June and July 2013, and 261 samples from soldiers returning from Kosovo, collected between March and April 2015. For comparison, 267 sera of soldiers prior to their missions, collected between April and September 2009, were included in the study. These soldiers had never been on a mission and their last vacation was at least 6 months ago. Sera were kept at − 20 °C until use.

### Study design

All patients were asked to fill out a detailed questionnaire. Demographic data, including age, sex and place of residence were collected. Additionally, information on the status of employment (professional soldier, militiamen), duration of the current mission, vacation during the mission, information on prior and on outdoor activities and animal contact during the mission was obtained. Data on *Leishmania* spp. seropositivity was available for the provided sera [15, 16].

### Seroprevalence

All 753 sera were tested for IgG antibodies by an indirect immunofluorescence test (Euroimmun, Lübeck, Germany) with subsequent fluorescence microscopy, following the manufacturer’s directions. Serum samples were used at an initial dilution of 1:10. Positive samples were subsequently further diluted to assess titers of 1:20, 1:40, 1:80, 1:100, 1:320 and 1:1000, testing for IgG antibodies against TOSV, SFNV, SFSV and CYPV.

All IgG positive samples were additionally tested for IgM antibodies against TOSV, SFNV, SFSV and CYPV, by the indirect immunofluorescence test described above. To avoid false results by interfering IgG antibodies, samples were incubated with EUROSORB (Euroimmun, Lübeck, Germany) for 15 min, subsequently centrifuged for 5 min at 2000× *rpm* and the supernatant was used for further testing, following the manufacturer’s directions as described above.

### Statistical analysis

Numerical data are presented as the mean and standard deviation (SD). A Mann-Whitney U-test was performed to evaluate differences between operational areas. Categorical data was analysed by a Fisher’s exact test, using *Phlebovirus* seropositivity as a predictor variable. Odds ratios (OR) with exact 95% confidence intervals (CI) were estimated. A two-sided *P*-value < 0.05 was considered statistically significant. All positive samples were included to calculate seroprevalences. Titers of 1:10 were excluded from the analysis to test risk factors associated with *Phlebovirus* seropositivity, as duration of mission and risk of infection by season. The risk of transmission was calculated based on the presence of a soldier during sand fly activity in the respective operational area. Presence during June, July and August was rated as high infection risk. Presence during two of the three months was rated as medium risk and presence during one or none of the three summer months was rated as low risk. Microsoft Excel 2011 for Mac and the R environment for Mac were used for data analysis.

## Results

### Seroprevalences

Altogether, 119 sera (15.8%, 119/753) showed IgG antibodies against phleboviruses. All 6 female soldiers included in the study were negative. No significant difference was observed between professional soldiers and militiamen (OR: 1.2, 95% CI: 0.8–1.8%, *P *= 0.48). Soldiers tested after their missions showed a significantly higher seroprevalence (18.1%, 88/486), than soldiers tested prior to their missions (11.6%, 31/267; OR: 1.7, 95% CI: 1.1–2.8%, *P *= 0.02). No significant differences were observed between the age groups. Seropositivity was observed in all age groups of soldiers returning from operational areas. In soldiers investigated prior to their missions only the age groups < 30 and 30–39 showed seropositivity, however sample sizes of older age groups were too low for statistical analysis (Table 1).

**Table 1:** Seroprevalences by age

	Age group (years)	Positive/Total (%)	OR	95% CI	*P*-value
Operational areas	< 30	33/252 (13.1)	Reference		
	30–39	8/95 (8.4)	0.6	0.2–1.4	0.2
	40–49	16/99 (16.2)	1.3	0.6–2.5	0.5
	> 50	4/40 (10.0)	0.7	0.2–2.0	0.6
Without prior mission	< 30	10/232 (4.3)	Reference		
	30–39	2/26 (7.7)	1.8	0.2–9.1	0.4
	40–49	0/7 (0)	–	–	–
	> 50	0/2 (0)	–	–	–

Of the 119 IgG positive sera, 30 (25.2%, 30/119) also showed IgM antibodies against phleboviruses. Soldiers tested after their missions showed a lower IgM seroprevalence (22.7%, 20/88), than soldiers tested prior to their missions (32.3%, 10/31; OR: 0.6, 95% CI: 0.2–1.7%, *P *= 0.39) (Table 2).

**Table 2:** Titers by operational areas

		1:10	1:20	1:40	1:80	1:100	1:320	1:1000	Total
IgG	Without prior mission	8/267 (2.99%)	3/267 (1.12%)	4/267 (1.50%)	4/267 (1.50%)	3/267 (1.12%)	9/267 (3.37%)	–	31/267 (11.6%)
	BIH	–	1/61 (1.64%)	1/61 (1.64%)	2/61 (3.28%)	3/ 61 (4.92%)	–	–	7/61 (11.47%)
	Kosovo	23/261 (8.81%)	3/261 (1.15%)	11/261 (4.21%)	3/261 (1.15%)	6/261 (2.30%)	7/261 (2.68%)	1/261 (0.38%)	54/261 (20.69%)
	Syria	3/101 (2.97%)	2/101 (1.98%)	4/101 (3.96%)	1/101 (0.99%)	3/ 101 (2.97%)	5/101 (4.95%)	–	18/101 (17.82%)
	Lebanon	1/63 (1.59%)	1/63 (1.59%)	–	1/63 (1.59%)	3/63 (4.76%)	–	3/63 (4.76%)	9/63 (14.28%)
IgM^a^	Without prior mission	2/31 (6.45%)	3/31 (9.68%)	3/31 (9.68%)		2/31 (6.45%)	–	–	10/31 (32.25%)
	BIH	–	–	–	–	–	–	–	0/7 (0.00%)
	Kosovo	5/54 (9.26%)	5/54 (9.26%)	–	2/54 (3.70%)	4/54 (7.41%)	–	–	16/54 (29.63%)
	Syria	–	1/18 (5.55%)	–	1/18 (5.55%)	1/18 (5.55%)	–	–	3/18 (16.67%)
	Lebanon	–	1/9 (11.11%)	–	–	–	–	–	1/9 (11.11%)

^a^Only IgG positive samples tested

### Geographical differences

Seroprevalences varied between the operational areas, being highest in soldiers returning from Kosovo (20.7%, 54/261), followed by Syria (17.8%, 18/101), Lebanon (14.3%, 9/63) and BIH (11.5%, 7/61) (Table 3).

**Table 3:** Seroprevalences by operational areas

	Titer ≥ 1:10		Titer ≥ 1:20		Titer ≥ 1:100	
	IgG positive/Total (%)	OR/95% CI (*P*-value)	IgG positive/Total (%)	OR/95% CI (*P*-value)	IgG positive/Total (%)	OR/95% CI (*P*-value)
Soldiers before missions	31/267 (11.6)	Reference	23/267 (8.6%)	Reference	12/267 (4.5)	Reference
BIH	7/61 (11.5)	1.0/0.4–2.5 (1.0)	7/61 (11.5%)	1.4/0.5–3.5 (0.46)	3/61 (4.9)	1.1/0.2–4.3 (1.0)
Kosovo	54/261 (20.7)	1.9/1.2–3.2 (0.006)*	31/261 (11.9%)	1.5/0.8–2.7 (0.25)	14/261 (5.4)	1.2/0.5–2.9 (0.70)
Syria	18/101 (17.8)	1.7/0.8–3.2 (0.12)	15/101 (14.9%)	2.0/0.9–4.3 (0.05)*	8/101 (7.9)	1.8/0.6–5.0 (0.20)
Lebanon	9/63 (14.3)	1.3/0.5–3.0 (0.50)	8/63 (12.7)	1.7/0.6–4.2 (0.22)	6/63 (9.5)	2.23/0.7–6.8 (0.13)

* Significant result (*P* < 0.05)

Highest rates of IgM seropositivity were observed in soldiers returning from Kosovo (29.6%, 16/54), followed by Syria (16.7%, 3/18) and Lebanon (11.1%, 1/9). No IgM titers were detected in soldiers returning from BIH (Table 2).

### Titers

IgG titers ranged from 1:10 to 1:1000 in soldiers returning from a mission, being highest in soldiers returning from Kosovo and Lebanon (1:1000), followed by Syria (1:320) and BIH (1:100). IgG titers in soldiers who had never been on a mission ranged from 1:10 to 1:320 (Table 2).

IgM titers ranged from 1:10 to 1:100 in soldiers returning from a mission, being highest in soldiers returning from Kosovo and Syria (1:100). IgM titers in soldiers who had not been on a mission also ranged from 1:10 to 1:100 (Table 2).

### Risk factors

In soldiers returning from operational areas, a significant correlation was observed between *Phlebovirus* seropositivity and at least one of the following symptoms: febrile illness, fever, nausea, malaise, limb and joint pain (OR: 1.9, 95% CI: 1.1–3.4, *P *= 0.03). Moreover, a significant correlation between *Phlebovirus* seropositivity and having had an episode of fever during the mission was observed (OR: 2.3, 95% CI: 1.03–4.6, *P *= 0.03) (Table 4). Also, there was a significant correlation between anti-*Phlebovirus* antibodies and anti-*Leishmania* antibodies (OR: 2.7, 95% CI: 1.2–5.8, *P *= 0.009) (Table 4).

**Table 4:** Risk factors for *Phlebovirus* seropositivity during a mission

Risk factors	OR	95% CI	*P*-value
Missions			
Prior mission	0.80	0.5–1.4	0.50
Kosovo	0.70	0.3–1.3	0.29
Syria	1.22	0.6–2.7	0.60
Bosnia	1.33	0.5–2.9	0.53
Symptoms			
Symptoms	1.90	1.1–3.4	0.03*
Fever	2.30	1.03–4.6	0.03*
Limb pain	1.03	0.3–3.1	1
Vacation	1.88	0.9–4.1	0.12
Outdoor activities			
Sports	1.40	0.5–4.6	0.70
Running	1.90	0.9–4.4	0.08^a^
Mountaineering	1.17	0.6–2.1	0.60
Cycling	0.97	0.5–2.0	1.0
Football	1.00	0.1–4.5	1.0
Animal contact			
Dog	1.10	0.6–2.0	0.80
Cat	1.40	0.7–2.6	0.27
Rodent	1.40	0.03–12.8	0.50
Pets	1.12	0.7–2.2	0.50
*Leishmania* spp. ELISA			
*Leishmania* antibodies	1.50	0.7–3.0	0.20
*Leishmania* positive	2.70	1.2–5.8	0.009*
*Leishmania* borderline	0.60	0.1–2.0	0.60

* Significant result (*P* < 0.05)

^a^Strong trend

A strong trend towards a correlation was observed between anti-*Phlebovirus* antibodies and the outdoor activity “running” during the respective mission (OR: 1.9, 95% CI: 0.9–4.4, *P *= 0.08) (Table 4). Seropositivity in soldiers having been on a mission for more than 11 months was more than double compared to soldiers having been on a mission for less than six months (OR: 2.5, 95% CI: 0.9–7.5, *P *= 0.07) (Table 5). Finally, seropositivity increased by 1.7-fold from low transmission risk (mission mainly not during summer) to high transmission risk (mission mainly during summer) (Table 6).

**Table 5:** Seroprevalence by duration of mission

Duration (months)	Total	IgG positive (%)	OR	95% CI	*P*-value
< 6	84	7 (8.33)	Reference		
6–11	315	38 (12.06)	1.5	0.6–4.2	0.44
> 11	87	16 (18.39)	2.5	0.9–7.5	0.07

**Table 6:** Seropositivity by risk of infection (sand fly activity)

Risk of infection	Total	IgG positive (%)	OR	95% CI	*P*-value
Low	275	32 (11.64)	Reference		
Medium	116	12 (10.34)	0.9	0.4–1.8	0.90
High	95	17 (17.89)	1.7	0.8–3.3	0.16

*Note*: risk of infection calculated: high, June, July and August; medium, two of three (June, July, August); low, one of three (June, July, August) and other months

### *Phlebovirus* serotypes

Although *Phlebovirus* diversity was not the focus of this study and the discriminatory power of the indirect immunofluorescence test is known to be low, the results for the different *Phlebovirus* serotypes are included for completeness. Generally, TOSV was always the most prevalent serotype (Table 7).

**Table 7:** Seroprevalences of *Phlebovirus* serotypes

		SFNV complex		SFSV complex	
		TOSV	SFNV	SFSV	CYPV
IgG	Without prior mission	22/31 (71.0%)	2/31 (6.5%)	7/31 (22.5%)	–
	Operational areas	45/88 (51.1%)	2/88 (2.3%)	39/88 (44.3%)	2/88 (2.3%)
IgM	Without prior mission	10/10^a^ (100%)	–	–	–
	Operational areas	13/20^a^ (65.0%)	–	7/20^a^ (35.0%)	–

^a^Percentage of the tested IgG positive samples

IgG antibodies against all four *Phlebovirus* serotypes were detected in soldiers returning from a mission and against three seroptypes (TOSV, SFNV and SFSV) in soldiers who had not been on a mission. While IgG antibodies against TOSV and SFSV were found in soldiers from all operational areas, IgG antibodies against CYPV were only found in soldiers returning from BIH. IgG antibodies against SFNV were found in one sample each in soldiers returning from Syria and Lebanon, respectively.

IgM antibodies against TOSV and SFSV were observed in soldiers returning from missions, while only TOSV was observed in soldiers who had not been on a mission.

## Discussion

This is the first study on *Phlebovirus* seroprevalence in Austrian military personnel and, to the best of our knowledge, in central Europe in general. We found unexpectedly high seroprevalences in soldiers returning from various missions abroad, whereby percentages of seropositivity varied between the operational areas. Interestingly, seropositivity was significantly associated with having had an episode of febrile illness during the mission. Also, the presence of anti-*Phlebovirus* antibodies significantly correlated with the presence of anti-*Leishmania* antibodies. Moreover, *Phlebovirus* infections were associated with longer missions, missions during sand fly season and the outdoor activity “running” during the mission.

Although, anti-*Phlebovirus* antibodies were found in all groups tested, the percentage of seropositivity differed considerably between the geographical areas, even between geographically nearby areas, such as BIH and Kosovo, revealing 11.5% and 20.7% seropositivity, respectively. This is in good accordance with the literature. For BIH, Hukic & Salimovic-Besic reported rather constant TOSV seroprevalences of 12.5%, 9.38%, 10.71% in 2006, 2007 and 2008 respectively [17]. For Kosovo, a SFSV seroprevalence of 9.6% was reported in 1976 [1] and a TOSV seroprevalence of 5.5% in 2011 [18], but in a recent study, a SFSV seroprevalence of up to 78.2% and a TOSV-seroprevalence of up to 11% was found in livestock [19].

To the best of our knowledge, the present study provides the first data on *Phlebovirus* epidemiology involving Syria and Lebanon, however, the circulation of sand fly fever viruses has been reported from the bordering countries Turkey and Iraq [20, 21]. The presence of sand flies in Syria and Lebanon, of course, is evident by leishmaniasis being highly endemic in this region [22].

Seroprevalences in soldiers returning from a mission did not increase with age, as has been reported for *Phlebovirus*-endemic countries [23–25]. Seroprevalence, however, is probably rather linked to the time of having been exposed to infected sand flies than age, which would fit well to our observation that seropositivity correlated to the length of the respective missions. Typically, Old World sand flies are active between spring and autumn, with either one or two peaks of activity. A longer duration of the mission not only increases the chance of being bitten by sand flies, but a duration of more than 11 months also grants to include at least one sand fly activity peak. In British military personnel deployed to Afghanistan, increased infection rates were observed during the summer months [13]. In military personnel, there generally is a trend towards age-independent seroprevalences of vector-borne diseases [14].

The fact that relatively high seroprevalences, albeit at lower titers, were also found in soldiers tested prior to their missions gives rise to the question whether autochthonous infections can be acquired in Austria. Austria, a central European country, has recently been proven to have stable sand fly populations [26–28]; however, until now only the species *P. mascittii* has been found and the vectorial capacity of *P. mascittii* for phleboviruses has not yet been investigated. Although there have been singular reports of supposedly autochthonous *Phlebovirus* infections from other central European countries, we assume that the seropositivity rather reflects the overall very high travel activity of the Austrian population and of soldiers in particular. As Austrians typically spend their summer holidays in the nearby Mediterranean countries and as military personnel participating in missions abroad typically show an overall increased travel activity, the soldiers can be expected to have already travelled to several *Phlebovirus* endemic regions in their lives [15]. Thus, as they however, reported to have not been outside of Austria in the past six months, the fact that a considerable percentage of them was seropositive rather corroborates the known longevity of anti-*Phlebovirus* antibodies than indicating autochthonous infections in Austria. Infections with phleboviruses are assumed to cause a lifelong immunity against the respective *Phlebovirus* serotype [7].

Interestingly, a significant association between seropositivity and having had at least one symptom of a febrile illness during the mission was observed, the rates of symptomatically infected soldiers varying between the operational areas. While only 11.8% of seropositive soldiers returning from BIH reported symptoms such as febrile illness, fever, nausea, malaise, limb and joint pain, 30.6% of the soldiers returning from Syria reported these symptoms. The data from the literature are controversial. Only one of nine *Phlebovirus* seropositive U.S. soldiers returning from Iraq reported a febrile illness [29]. Also, the majority of seropositive Iranian soldiers stationed at the Western border of Iran did not report any symptoms [14]. However, up to 70% of symptomatic Pakistani military personnel and 92.9% of US Army soldiers operating in Iraq reported an uncertain febrile illness with various symptoms, including high fever, headache, myalgia, malaise and severe performance reduction of up to one week [12, 30]. Together with our findings, these reports highlight the diversity of etiopathologies of *Phlebovirus* infections.

A significant association was observed between *Phlebovirus* seropositivity and *Leishmania* seropositivity. Co-infections can be acquired if suitable sand fly species and both pathogens are present. *Leishmania* spp. are endemic in Kosovo, Syria and Lebanon [22, 31] and also vector-competent sand fly species, such as *Phlebotomus papatasi* and *P. tobbi*, occur in the investigated operational areas [32–34]. Similarly to *Phlebovirus* infections, *Leishmania* infections are often associated to unspecific symptoms and thus remain undiagnosed [16].

A clear trend was observed between the outdoor activity “running” and *Phlebovirus* seropositivity. During missions, the time for sports typically is the late afternoon and early evening, when temperatures drop to moderate levels in the respective regions. This coincides with the nocturnal activity of most sand fly species, increasing with sunset and decreasing with sunrise [35]. Moreover, running is typically associated with heavy sweating and high CO_2_ exhalation, thus, during stretching phases after the run, joggers are ideal blood-meal hosts for blood-sucking insects. Heavy sweating also leads to a reduced protection by repellents, if used at all. Reduced protection by repellents was for example reported for US soldiers deployed in Iraq as a result of heavy sweating during sleep [36]. The attraction of sand flies to CO_2_ and human odor could contribute to increased biting rates after running, as also assumed by van Thiel et al. [37], who observed an association between off-duty sports during late afternoon and sand fly attack rates. Finally, for off-duty activities, military personnel typically wear shorts and T-shirts instead of their long-sleeved uniforms.

The screening for anti-CYPV and anti-SFNV antibodies revealed that these two virus types both play a minor role in the areas tested. However, CYPV and SFNV are genetically rather similar to SFSV and TOSV, respectively, and thus not reliably distinguishable by serology. CYPV, discovered rather recently, has been isolated from febrile Greek soldiers in Cyprus [11]. The two anti-CYPV antibody positive sera of soldiers returning from BIH detected in the current study are assumed to be most likely the result of an infection acquired during a previous vacation in Cyprus.

This study has several limitations. First, due to logistic issues of the Austrian Armed Forces, either only sera obtained after or before a mission and not both were available for the soldiers participating in this study. Secondly, the retrospective design of the study does not allow to faultlessly conclude that all *Phlebovirus* infections were indeed acquired during the respective mission. Low titers, particularly, could be the result of infections acquired on previous missions or also during holiday travels. However, in many cases and from all operational areas relatively high titers were detected and often the assumption of a recent infection was also supported by detectable IgM titers. Finally, as mentioned before, the IIFT is susceptible to cross-reactions, which could impair the reliability of the results concerning *Phlebovirus* serotypes, but this does not affect the overall seroprevalences.

## Conclusions

This study clearly indicates that soldiers are exposed to sand flies and are at significant risk to infection with phleboviruses during missions in southern Europe and the Middle East. Thus, *Phlebovirus* infections should be considered as differential diagnosis when military personnel show unspecific febrile symptoms during deployment in endemic areas and adequate prevention measures should be taken, including protective clothing and the usage of repellents during evening outdoor activities and fine-meshed impregnated bed nets while sleeping. Moreover, the observed relatively high seropositivity in soldiers without previous mission indicates that also frequent vacation trips to endemic areas pose a considerable risk to acquire a *Phlebovirus* infection.

## Data Availability

All data generated or analysed during this study are included in this article.

## Abbreviations

BIH: Bosnia and Herzegovina
IIFT: Indirect-immunofluorescence test
TOSV: Toscana virus
SFNV: Sand fly fever Naples phlebovirus
SFSV: Sand fly fever Sicilian virus
CYPV: Cyprus virus
SD: standard deviation
OR: odds ratio
CI: confidence interval
